# Evaluating Treatment Outcomes of Vitamin B12 and Folic Acid Supplementation in Obsessive-Compulsive Disorder Patients With Deficiencies: A Comparative Analysis

**DOI:** 10.7759/cureus.82420

**Published:** 2025-04-17

**Authors:** Sultana Algin, Tanbir Ahmed, Md Munaim Reza, Asha Akter, Nusrat Jahan Tanzilla, Md. Ahsanul Haq, Rahnuma Ahmad, Miral Mehta, Mainul Haque

**Affiliations:** 1 Department of Psychiatry, Bangabandhu Sheikh Mujib Medical University, Dhaka, BGD; 2 Department of Psychiatry, Enam Medical College and Hospital, Savar, BGD; 3 Department of Biostatistics, International Centre for Diarrhoeal Disease Research, Bangladesh (icddr,b), Dhaka, BGD; 4 Department of Physiology, Medical College for Women and Hospital, Dhaka, BGD; 5 Department of Pedodontics and Preventive Dentistry, Karnavati School of Dentistry, Karnavati University, Gandhinagar, IND; 6 Department of Pharmacology and Therapeutics, National Defence University of Malaysia, Kuala Lumpur, MYS; 7 Department of Research, Karnavati School of Dentistry, Karnavati University, Gandhinagar, IND

**Keywords:** biochemical markers of ocd, comparative study, folic acid, homocysteine, micronutrient deficiency, neuropsychiatric disorder, nutritional supplementation, obsessive-compulsive disorder treatment, one-carbon metabolism, vitamin b12

## Abstract

Introduction

Obsessive-compulsive disorder (OCD) imposes a considerable impact on day-to-day functioning. Many people experience insufficient symptom relief even after taking the optimum dose of OCD medications. Reduced levels of folic acid and vitamin B₁₂, along with elevated homocysteine (HCY), have been suggested as possible factors in the persistence of obsessive-compulsive (OC) symptoms. This study investigated how supplementation of vitamin B₁₂, folic acid, and selective serotonin reuptake inhibitors (SSRIs) affects OC symptoms and related biochemical markers.

Methods

A comparative study enrolled 72 OCD patients. For eight weeks, the conventional treatment group received SSRIs or other anti-obsessive medication. In contrast, the nutrient-supplemented group received supplements of vitamin B₁₂, folic acid, and SSRIs. Micronutrients HCY, folic acid, and vitamin B₁₂ were measured at baseline and after eight weeks. Besides, the Yale-Brown Obsessive Compulsive Scale (Y-BOCS) was applied to assess the severity of OCD symptoms at the baseline, four-week, and eight-week visits.

Results

Group A (conventional treatment with nutrient supplement) showed significant improvements in vitamin B₁₂, blood folic acid, and reductions in HCY levels compared to Group B (conventional treatment). However, no substantial differences in insight levels were observed between the groups. Both groups exhibited decreased Y-BOCS scores, indicating a reduction in OCD symptoms; however, the improvements in Group A (conventional treatment + nutrient supplement) were statistically significant.

Conclusions

When taken with SSRIs, vitamin B₁₂and folic acid supplements seem to improve OCD patients’ clinical results. These results imply that this supplementation could be a useful therapeutic adjunct.

## Introduction

WHO has listed obsessive-compulsive disorder (OCD) as one of the top 10 most disabling disorders. Individuals with OCD frequently experience substantial distress, which is used to avoid circumstances that cause difficulty. This avoidance results in fewer social interactions and a decreased quality of life. Unfortunately, many OCD patients suffer silently without receiving a diagnosis. It gets harder to control if the disease is left untreated, as the brain undergoes structural changes [1,2]. OCD is a complicated condition that is impacted by environmental and genetic variables [3]. It can deteriorate over time and result in increasingly severe clinical effects if treatment is not received [4]. However, only a few patients receive the appropriate care, and clinicians frequently misdiagnose and undertreat it. Uncertainty surrounding the biology of OCD has impeded efforts to create more potent therapies [5].

A typical activity in brain regions affects neurotransmitter metabolism, whose synthesis and breakdown are closely linked to one-carbon metabolism, and OCD impairments may be linked to corticostriatal system dysfunction [6]. Numerous studies have recently discovered that vitamin B₁₂, folate, and homocysteine (HCY) levels in one-carbon metabolism may be linked to some mental health problems [7-9]. These methylation and metabolic mechanisms might be crucial in the emergence of neuropsychiatric symptoms, such as obsessive-compulsive (OC) symptoms [6,10], where vitamin B₁₂ helps in methylating proteins, neurotransmitters, and phospholipids in nerve cells [6,8]. Folate also helps by donating methyl groups, converting HCY into methionine, which lowers HCY levels and supports proper methylation and neurotransmitter production [8,11].

A meta-analysis has highlighted the importance of folic acid and vitamin B₁₂ in maintaining neuropsychiatric health. Deficiency in vitamin B₁₂ affects 4-50% of patients with neuropsychiatric symptoms. Both vitamin B₁₂ and folic acid are increasingly used in psychiatric treatments. Their interaction with HCY, genetic factors, and neurotransmitter metabolism is particularly relevant for conditions like OCD [12]. Mental symptoms, particularly mood and psychotic illnesses, have been associated with levels of HCY, folic acid, and vitamin B₁₂. The synthesis of neurotransmitters like serotonin and catecholamines depends on carbon transfer metabolism (methylation), which is mediated mainly by these nutrients [13]. While the effects of folic acid and B₁₂ deficiencies, disruptions in one-carbon metabolism, and the use of folic acid and its derivatives as antidepressants or to enhance antidepressant action have been well-studied in depression, similar research has not been conducted regarding OCD [9].

Nutritional supplements have been used in psychiatric disorders like OCD [8,9], with vitamin B benefiting schizophrenia [14], folic acid aiding depression [15], and both vitamin B₁₂ and folic acid improving postpartum depression [16]. Despite mounting research, the impact of vitamin B₁₂ on mental health conditions remains unclear [16]. However, the effect of vitamin B₁₂ and folic acid supplementation in the treatment of OCD has not been evaluated much. One randomized, double-blind control study in Turkey [9] assessed the effect of adding folate to fluoxetine in the treatment of OCD. This study reported no significant improvement over the control group. However, high HCY levels, folic acid, and vitamin B₁₂ deficiencies are frequently linked to treatment-resistant OCD [7,12]. These findings indicate that metabolic abnormalities in these nutrients may contribute to the onset and persistence of OCD symptoms.

The limited effectiveness and possible side effects of existing drugs, including selective serotonin reuptake inhibitors, serotonin noradrenaline reuptake inhibitors, and the tricyclic antidepressant clomipramine, have led to a search for alternative strategies. It is commonly known that a range of nutrient deficiencies can be seen in patients with mental problems. Research suggests that vitamin B₁₂, folic acid, and HCY may be connected to the development of OCD. Consequently, vitamin B₁₂ and folic acid are thought to help manage OCD [8]. The lack of a thorough understanding of OCD’s underlying origins has impeded the development of novel therapies. Vitamin B₁₂ and folic acid deficiencies may contribute to low monoamine synthesis in OCD patients, further affecting their symptoms.

This study aimed to measure vitamin B₁₂, folic acid, and HCY levels to explore whether changes in these levels influence the progression of OCD and how micronutrient supplementation might impact those with deficiencies/imbalances in these nutrients and their OC symptoms over time.

Problem statement

OCD is a chronic mental illness characterized by distressing, intrusive thoughts and repetitive behavior [17]. Despite tremendous progress in management, the precise cause of OCD and the factors affecting its development are not entirely understood yet [18]. One area of particular interest in the pathophysiology of OCD is the potential role of micronutrients, such as vitamin B₁₂, folic acid, and HCY [12,13,17,19]. In particular, the study will look into how a supplement influences key biomarkers such as serum folic acid, vitamin B₁₂, and HCY levels and how these changes relate to improvements in OCD. Despite the availability of treatments for OCD, there is currently a dearth of evidence about the potential metabolic and psychological benefits of therapy, particularly about quality of life and long-term symptom management [20]. By assessing the effects of an intervention on biochemical markers and clinical parameters in OCD patients over eight weeks, the current study aims to close this gap [9,21].

Objectives

This comparative observational study aimed to assess serum levels of vitamin B₁₂, folic acid, and HCY in patients with OCD who presented with micronutrient abnormalities. Depending on individual deficiencies, patients received supplementation with vitamin B₁₂, folic acid, or both. The objectives were as follows: (i) to evaluate the effects of nutrient supplementation on biochemical markers - specifically serum vitamin B₁₂, folic acid, and HCY - and their relationship with the severity of OC symptoms and insight levels; (ii) to identify how metabolic abnormalities in these micronutrients may influence OCD outcomes; (iii) to determine whether supplementation improves biomarker levels and reduces OC symptom severity, as measured by the Yale-Brown Obsessive Compulsive Scale (Y-BOCS), at the fourth and eighth weeks of follow-up; (iv) to explore whether changes in biochemical markers correspond with changes in OCD symptoms; (v) to provide insights that could inform future research on the connection between metabolic or nutritional treatments and OCD symptomatology; and (vi) to examine the long-term impact of micronutrient-based interventions on both clinical and biochemical outcomes.

## Materials and methods

The investigation was conducted in two phases. The first phase was a cross-sectional descriptive study, which has already been completed [7]. The second phase was a comparative study carried out at the Department of Psychiatry, Bangabandhu Sheikh Mujib Medical University (BSMMU), Dhaka, Bangladesh, between January 2023 and June 2024. This study received approval from the Institutional Review Board (IRB) at BSMMU. Demographic data were collected using a semi-structured questionnaire, and written informed consent was obtained from all participants.

The clinical and sociodemographic questionnaire was developed based on previous research and adapted to the Bangladeshi context by a psychiatrist at BSMMU [7,22]. Participants were recruited from the specialized OCD clinic and the outpatient department of the Department of Psychiatry at BSMMU. After applying the inclusion and exclusion criteria, 72 patients were initially enrolled. However, due to loss to follow-up during the COVID-19 pandemic, 60 patients completed the study. These were evenly divided into two groups: 30 in the nutrient-supplemented group (Group A) and 30 in the conventional treatment group (Group B).

**Figure 1:**
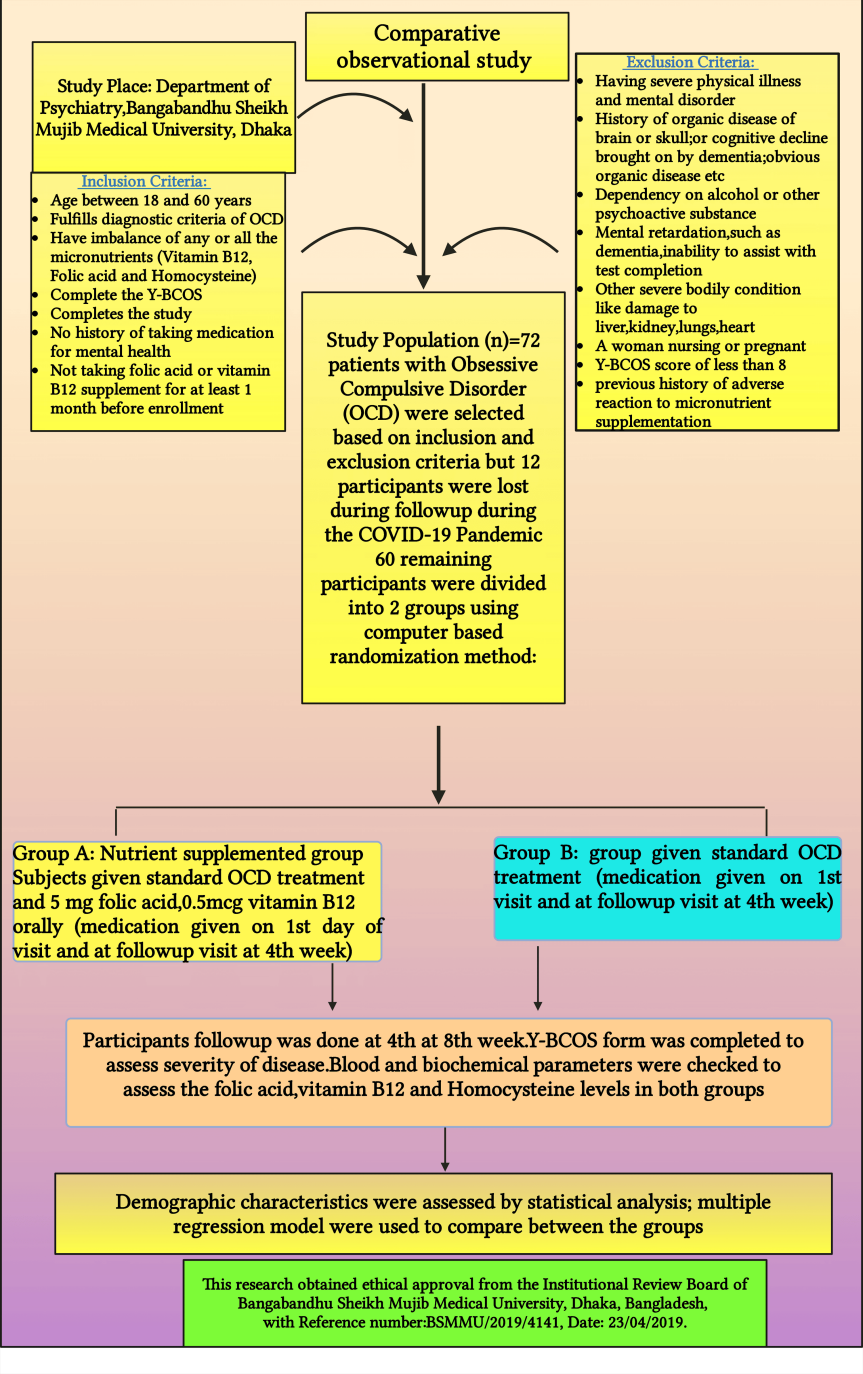
Flowchart illustrating the materials and methods employed in this study OCD, obsessive-compulsive disorder; Y-BOCS, Yale-Brown Obsessive Compulsive Scale The premium version of BioRender [23] (biorender.com) was used to create this figure, accessed on March 18, 2025, under license agreement number XJ281EM833. Illustration credit: Rahnuma Ahmadv

Group A received standard OCD treatment along with additional supplementation tailored to their specific micronutrient deficiencies. This included 5 mg/day of folic acid for low folate levels, 500 mcg/day of cyanocobalamin for vitamin B₁₂ deficiency, or both vitamins in the case of hyperhomocysteinemia. Group B received only standard OCD treatment (Figure 1) [23]. Group assignment was done using a computer-based randomization method. Patients in Group A received their medication and supplements in a pillbox, while Group B was provided only anti-obsessive medication at both the initial visit and the first follow-up at week 4.

Both groups were followed up at the fourth and eighth weeks, with additional telephone check-ins in between. Adherence and compliance were monitored through pill counts and discussions with both patients and caregivers. At each visit, potential side effects were reviewed, and physical examinations were conducted [22,24,25].

OCD severity was assessed using the Bangla-validated Y-BOCS [26] and the Clinical Global Impressions Severity Scale (CGI-S) at baseline, week 4, and week 8. Y-BOCS scores were interpreted as follows [7]: 0-7 (subclinical), 8-15 (mild), 16-23 (moderate), 24-31 (severe), and 32-40 (extreme). CGI-S ratings were 1 = normal, not at all ill; 2 = borderline mentally ill; 3 = mildly ill; 4 = moderately ill; 5 = markedly ill; 6 = severely ill; and 7 = among the most extremely ill patients [27].

Study subject selection strategy

The inclusion criteria for this study were as follows: participants had to be aged between 18 and 60 years, fulfill the Diagnostic and Statistical Manual of Mental Disorders, Fifth Edition criteria for OCD, and exhibit an imbalance in one or more micronutrients (folate, vitamin B₁₂, or HCY). Additionally, participants were required to complete the Y-BOCS and demonstrate the ability to complete the study. They also had to have no history of mental health medication use and were not allowed to take oral folic acid or vitamin B₁₂ at least one month prior to enrollment [7,9,10].

The exclusion criteria included individuals with severe physical illnesses or mental disorders such as schizophrenia, depressive disorder, panic disorder, or bipolar disorder. Those with a history of organic brain diseases, cognitive decline due to dementia, or other prominent organic diseases were also excluded. Furthermore, individuals with alcohol or psychoactive substance dependence, mental retardation, or conditions that hindered their ability to complete tests were excluded. Severe bodily conditions, including damage to the liver, kidneys, heart, or lungs, as well as pregnant or breastfeeding women, were not eligible for participation. Finally, participants with a Y-BOCS score lower than 8 or a history of adverse reactions to micronutrient intake were also excluded [7,9,10,13].

Blood sample collection

After an overnight fast, aseptic venous blood samples were collected. A total of 6 mL was drawn: 3-4 mL into a plain (red-top) tube for the analysis of folic acid and vitamin B₁₂, and 2-3 mL into a purple-top tube containing EDTA for HCY analysis. The samples were then centrifuged at 3,000 rpm for five minutes, and the serum and plasma were separated and stored in polythene tubes for future use [7].

Biochemical analysis

Serum vitamin B₁₂ was measured using a solid-phase, competitive chemiluminescent immunoassay. The serum level of folic acid was determined using a ligand-labeled, competitive chemiluminescent assay. Plasma HCY was calculated using a competitive chemiluminescent enzyme immunoassay [7]. The Immulite 1000 chemiluminescent analyzer performed all the tests at the Department of Biochemistry and Molecular Biology, BSMMU, Dhaka. Serum levels of vitamin B₁₂, folate, and HCY are detailed in Table 1 [13].

**Table 1:** Identification of micronutrient abnormalities based on cutoff values HCY, homocysteine Table credit: Tanbir Ahmed

Name	Cutoffs values
Serum vitamin B₁₂	<200 pg/mL (vitamin B₁₂ deficiency)
Serum folate level	<3 ng/mL (folate deficiency)
Plasma HCY	>14.0 μmol/L (hyperhomocysteinemia)

Ethical approval

All ethical considerations were addressed prior to the commencement of the study. The Institutional Review Board (IRB) at BSMMU, Dhaka, Bangladesh, granted ethical approval with reference number BSMMU/2019/4141 on April 23, 2019. Due to COVID-19, the research faced significant delays and was deferred for over two years. All participants provided written informed consent after being fully briefed on the study’s objectives, including the possibility of future publication. All other research-related ethical concerns were carefully considered and handled with the utmost attention.

Statistical analysis

Descriptive statistics were initially computed to compare baseline demographic and clinical characteristics between Group A (participants receiving conventional treatment plus nutrient supplementation) and Group B (participants receiving conventional treatment only). Differences between the groups were assessed using appropriate statistical tests (e.g., chi-square tests for categorical variables and t-tests for continuous variables) to ensure comparability at baseline.

Changes in biochemical markers - vitamin B₁₂ levels, serum folic acid, and HCY - were evaluated before and after the intervention to assess the effect of nutrient supplementation. A multivariate regression model was used to compare the post-intervention mean levels of these markers between the two groups while controlling for potential confounding factors, including age, sex, marital status, education level, place of residence (urban/rural), and occupation. This model allowed for the estimation of adjusted mean differences and corresponding p-values, providing a more accurate assessment of the intervention effect by accounting for baseline covariates. Statistical significance was determined using adjusted p-values derived from the model, with a significance threshold set at p < 0.05.

Associations between treatment groups were examined using the chi-square test for categorical clinical outcome measures, such as Y-BOCS score categories, insight level, and Clinical Global Impression Scale (CGIS) categories. For tables with more than a 2 × 2 contingency, Yates’s continuity correction was applied to minimize the risk of overestimating significance.

All statistical analyses were performed using STATA version 15 (StataCorp LLC, College Station, TX, USA). Graphical representations of results, including changes in biomarker levels and clinical scores, were generated using GraphPad Prism, Version 10.4.0 (GraphPad Software, LLC, San Diego, CA, USA). The results are presented with mean differences and 95% CIs to illustrate the magnitude and precision of the observed effects, providing a comprehensive evaluation of the impact of nutrient supplementation alongside conventional treatment.

## Results

Table 2 presents the demographic characteristics of Group A (conventional treatment + nutrient supplement) and Group B (traditional treatment). Both groups had similar mean ages (27.2 ± 8.78 vs. 27.3 ± 9.95 years). The proportion of males was higher in both groups, though slightly lower in Group B (80.0% vs. 70.0%). A larger percentage of participants in Group A were unmarried (76.7% vs. 63.3%), while Group B had a slightly higher proportion of married individuals (33.3% vs. 23.3%). Education levels differed between the groups, with more graduates in Group B (43.3% vs. 20.0%) and a higher proportion of participants with secondary education in Group A (50.0% vs. 33.3%). Both groups had similar occupational distributions, with students representing the largest subgroup. Most participants in both groups resided in urban areas (73.3% in Group A vs. 76.7% in Group B), and most identified as Muslim (86.7% vs. 90.0%). Group B had a higher proportion of nuclear families (70.0% vs. 56.7%), while birth order distribution was relatively balanced across the groups. Overall, the demographic characteristics were comparable between the two groups, with some variations in education, family patterns, and marital status.

**Table 2:** Demographic characteristics of Group A and Group B Data are presented as mean ± SD or as numbers with percentages in parentheses. ¹ Conventional treatment + nutrient supplement ² Conventional treatment only Table credit: Md. Ahsanul Haq

Independent observations	Group A¹ (n = 30)	Group B² (n = 30)
Age, years	27.2 ± 8.78	27.3 ± 9.95
Sex		
Male	24 (80.0%)	21 (70.0%)
Female	6 (20.0%)	9 (30.0%)
Marital status		
Unmarried	23 (76.7%)	19 (63.3%)
Married	7 (23.3%)	10 (33.3%)
Widowed/separated/divorced	0	1 (3.3%)
Education qualification		
Primary	8 (26.7%)	5 (16.7%)
Secondary	15 (50.0%)	10 (33.3%)
Graduate	6 (20.0%)	13 (43.3%)
Postgraduate	1 (3.3%)	2 (6.6%)
Occupation		
Unemployed	3 (10.0%)	4 (13.3%)
Student	16 (53.3%)	13 (43.3%)
Housewife	2 (6.7%)	5 (16.7%)
Service	7 (23.3%)	7 (23.3%)
Business	2 (6.7%)	1 (3.3%)
Residence		
Rural	8 (26.7%)	7 (23.3%)
Urban	22 (73.3%)	23 (76.7%)
Religion		
Muslim	26 (86.7%)	27 (90.0%)
Hindu	4 (13.3%)	3 (10.0%)
Family pattern		
Nuclear	17 (56.7%)	21 (70.0%)
Joint	13 (43.3%)	9 (30.0%)
Birth order		
First	8 (26.7%)	10 (33.3%)
Last	9 (30.0%)	10 (33.3%)
Others	13 (43.3%)	10 (33.3%)

**Figure 2:**
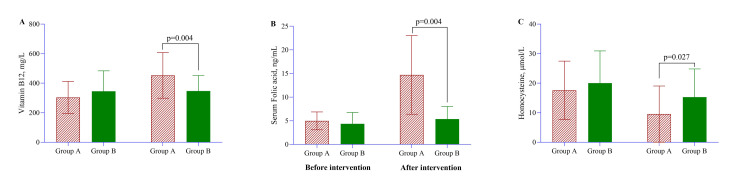
Treatment effect between Group A and Group B on vitamin B₁₂ (A), serum folic acid (B), and HCY (C) before and after the intervention period Data are presented as mean ± SD. The p-value was calculated using a multivariate regression model, adjusted for age, sex, marital status, education, residence, and occupation. HCY, homocysteine Illustration credit: Md. Ahsanul Haq

The treatment effect analysis comparing Group A (conventional treatment + nutrient supplement) and Group B (conventional treatment) on vitamin B₁₂, serum folic acid, and HCY levels before and after the intervention revealed significant differences post-intervention. While baseline vitamin B₁₂ levels were similar between the groups (p = 0.218), Group A showed a substantial increase in vitamin B₁₂ levels after the intervention (452.9 ± 154.4 mg/L vs. 347 ± 104.5 mg/L, p = 0.004) (Figure 2A). Similarly, serum folic acid levels improved significantly in Group A (14.7 ± 8.31 ng/mL) compared to Group B (5.36 ± 2.66 ng/mL, p < 0.001), despite no significant differences at baseline (p = 0.265) (Figure 2B). HCY levels, which were initially similar (p = 0.428), decreased significantly in Group A (9.54 ± 9.52 μmol/L) compared to Group B (15.3 ± 9.53 μmol/L, p = 0.027) post-intervention (Figure 2C). These results suggest that adding nutrient supplementation in Group A led to a significant improvement in vitamin B₁₂ and serum folic acid levels, while effectively reducing HCY concentrations, in comparison to conventional treatment alone.

The proportion of participants with Excellent insight was slightly higher in Group B (16.7%) compared to Group A (10.0%), while the Good and Fair insight categories were similarly distributed between the two groups. A p-value of 0.741 indicates that there was no statistically significant difference in insight levels between Group A (conventional treatment + nutrient supplement) and Group B (conventional treatment only) at week 8 following the intervention (Table 3).

**Table 3:** Association of insight levels between Group A and Group B after eight weeks of treatment Data are presented as numbers (percentages). The chi-square test with Yates’ continuity correction was applied for contingency tables larger than 2 × 2 to estimate the p-value. ¹ Conventional treatment + nutrient supplement ² Conventional treatment only Table credit: Md. Ahsanul Haq

Insight level category	Group A^1^ (n = 30)	Group B² (n = 30)	p-value
Excellent	3 (10.0%)	5 (16.7%)	0.741
Good	21 (70.0%)	19 (63.3%)	
Fair	6 (20.0%)	6 (20.0%)	

The proportion of participants classified as “normal” on the CGIS was identical between the two groups (36.7% in both Group A and Group B), and the distribution across other categories was also comparable. The p-value of 0.904 indicates no statistically significant difference in CGIS scores [27] between the groups after the intervention (Table 4).

**Table 4:** Association of CGIS scores between Group A and Group B after eight weeks of intervention Data are presented as numbers with percentages in parentheses. The chi-square test with Yates’ continuity correction was applied for contingency tables larger than 2 × 2 to estimate the p-value. ¹ Conventional treatment + nutrient supplement ² Conventional treatment only CGIS, Clinical Global Impression Scale Table credit: Md. Ahsanul Haq

CGIS score category	Group A¹ (n = 30)	Group B² (n = 30)	p-value
Normal	11 (36.7%)	11 (36.7%)	0.904
Borderline mentally ill	6 (20.0%)	8 (26.7%)	
Mildly ill	10 (33.3%)	9 (30.0%)	

At four weeks, the Y-BOCS severity categories showed that a higher proportion of participants in Group A exhibited mild symptoms compared to Group B (30.0% vs. 10.0%), while a greater percentage in Group B fell into the severe category (36.7% vs. 20.0%). The proportion of participants in the moderate category was similar in both groups (50.0% in Group A vs. 53.3% in Group B). However, the chi-square test with Yates’ continuity correction produced a p-value of 0.105, indicating that the observed differences in severity distribution were not statistically significant (Table 5).

**Table 5:** Association of Y-BOCS scores between Group A and Group B at four weeks Data are presented as numbers with percentages in parentheses. The chi-square test with Yates’ continuity correction was applied for contingency tables larger than 2 × 2 to estimate the p-value. ¹ Conventional treatment + nutrient supplement ² Conventional treatment only Y-BOCS, Yale-Brown Obsessive Compulsive Scale Table credit: Md. Ahsanul Haque

Y-BOCS score category	Group A¹ (n = 30)	Group B² (n = 30)	p-value
Mild (8-15)	9 (30.0%)	3 (10.0%)	0.105
Moderate (16-23)	15 (50.0%)	16 (53.3%)	
Severe (24-31)	6 (20.0%)	11 (36.7%)	

In Group A, 43.3% of participants exhibited mild symptoms compared to 16.7% in Group B, while the proportion of severe cases was lower in Group A (13.3% vs. 26.7% in Group B). The chi-square test with Yates' continuity correction yielded a p-value of 0.046 (Table 6), indicating a statistically significant difference in symptom severity between the groups. These findings suggest that adding a nutrient supplement to conventional treatment may enhance the reduction of OCD symptoms.

**Table 6:** Association of Y-BOCS scores between Group A and Group B at eight weeks Data are presented as numbers with percentages in parentheses. The chi-square test with Yates’ continuity correction was applied for contingency tables larger than 2 × 2 to estimate the p-value. ¹ Conventional treatment + nutrient supplement ² Conventional treatment only Y-BOCS, Yale-Brown Obsessive Compulsive Scale

Y-BOCS score category	Group A¹ (n = 30)	Group B² (n = 30)	p-value
Mild (8-15)	13 (43.3%)	5 (16.7%)	0.046
Moderate (16-23)	13 (43.3%)	17 (56.7%)	
Severe (24-31)	4 (13.3%)	8 (26.7%)	

## Discussion

We explored the relationship between vitamin B₁₂, folic acid, and blood HCY imbalances in OCD patients receiving micronutrient supplements. Our findings suggest that the administration of folic acid and vitamin B₁₂ positively mediated the association between OC symptoms and serum micronutrient levels. The sample was predominantly composed of young adult males (75% male, n = 45; 25% female, n = 15). Notably, the gender distribution varied significantly between the treatment groups, with a male preponderance in many areas. This pattern aligns with global trends, which indicate a higher prevalence of OCD among males.

**Figure 3:**
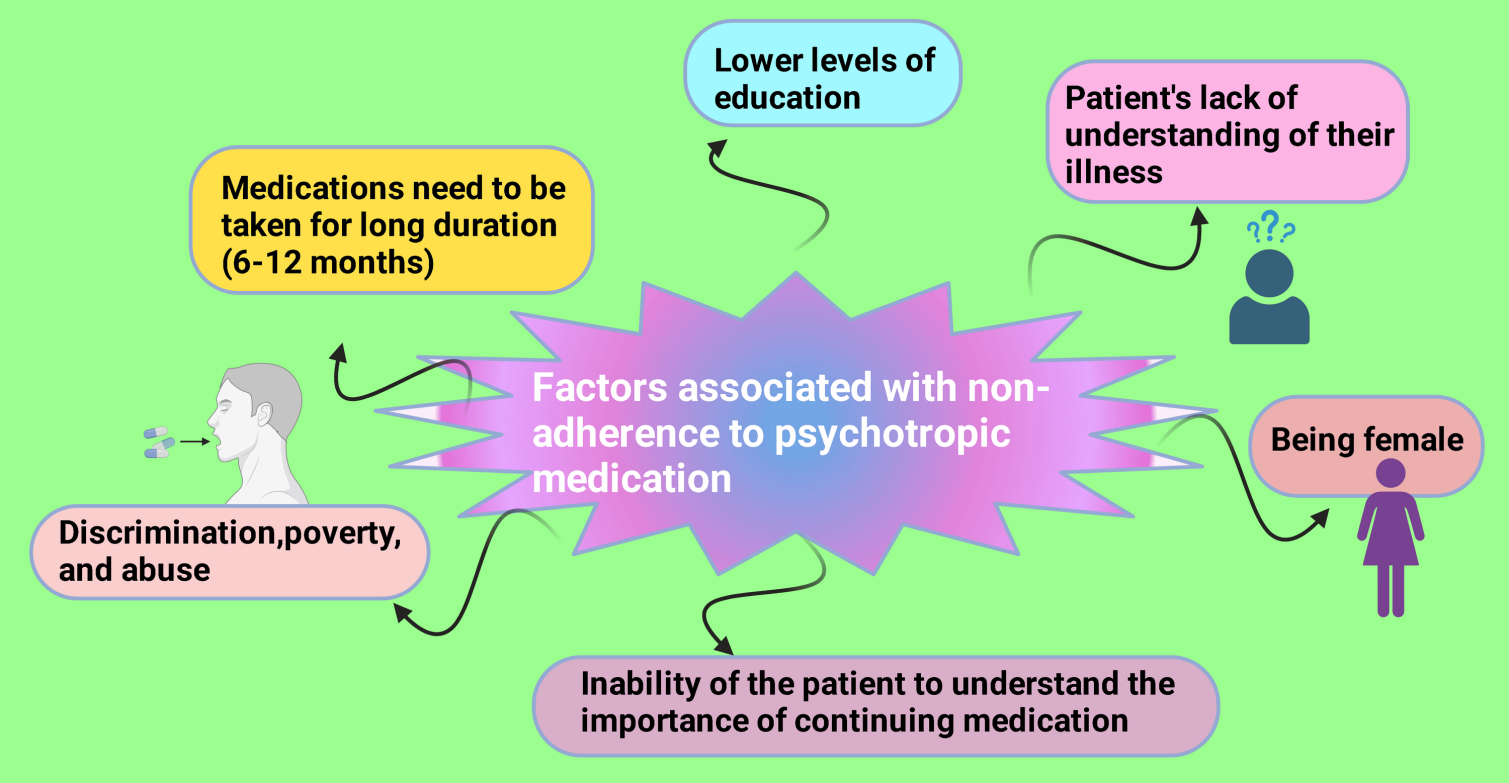
Factors associated with nonadherence to psychotropic medication The premium version of BioRender [23] (biorender.com) was used to create this figure, accessed on March 18, 2025, under license agreement number BY281GFESY. Illustration credit: Rahnuma Ahmad

With a population of 163 million, Bangladesh is a low-middle-income country facing several challenges within its healthcare system. The country faces the foremost obstacles to healthcare, with 74% of its population being literate and 63% living in rural areas [28]. These include a lack of knowledge about illnesses, inadequate communication between patients and medical professionals, restricted access to high-quality care and prescription drugs, lengthy wait periods at medical facilities, and lengthy clinic commutes [29]. From a socioeconomic perspective, marginalized women in Bangladesh are especially vulnerable to discrimination, poverty, and abuse. Women living in rural and impoverished areas face the harshest conditions, and their struggles are intensified by the nation's political and economic instability (Figure 3) [30].

Additionally, psychiatric patients who had less education in secondary school are more likely than those with more outstanding education to not take their psychotropic medication as prescribed. Being a woman was also found to be associated with medication failure to comply. Patients’ lack of insight or understanding of their illness and the importance of their medication was also a common factor contributing to nonadherence to psychotropic medication. Another factor linked to medication nonparticipation was the length of treatment [31]. Vitamin B₁₂, serum folic acid, and HCY levels were significantly different between Group A (conventional treatment + nutrient supplement) and Group B (conventional treatment) after the intervention, according to the treatment effects analysis.

**Figure 4:**
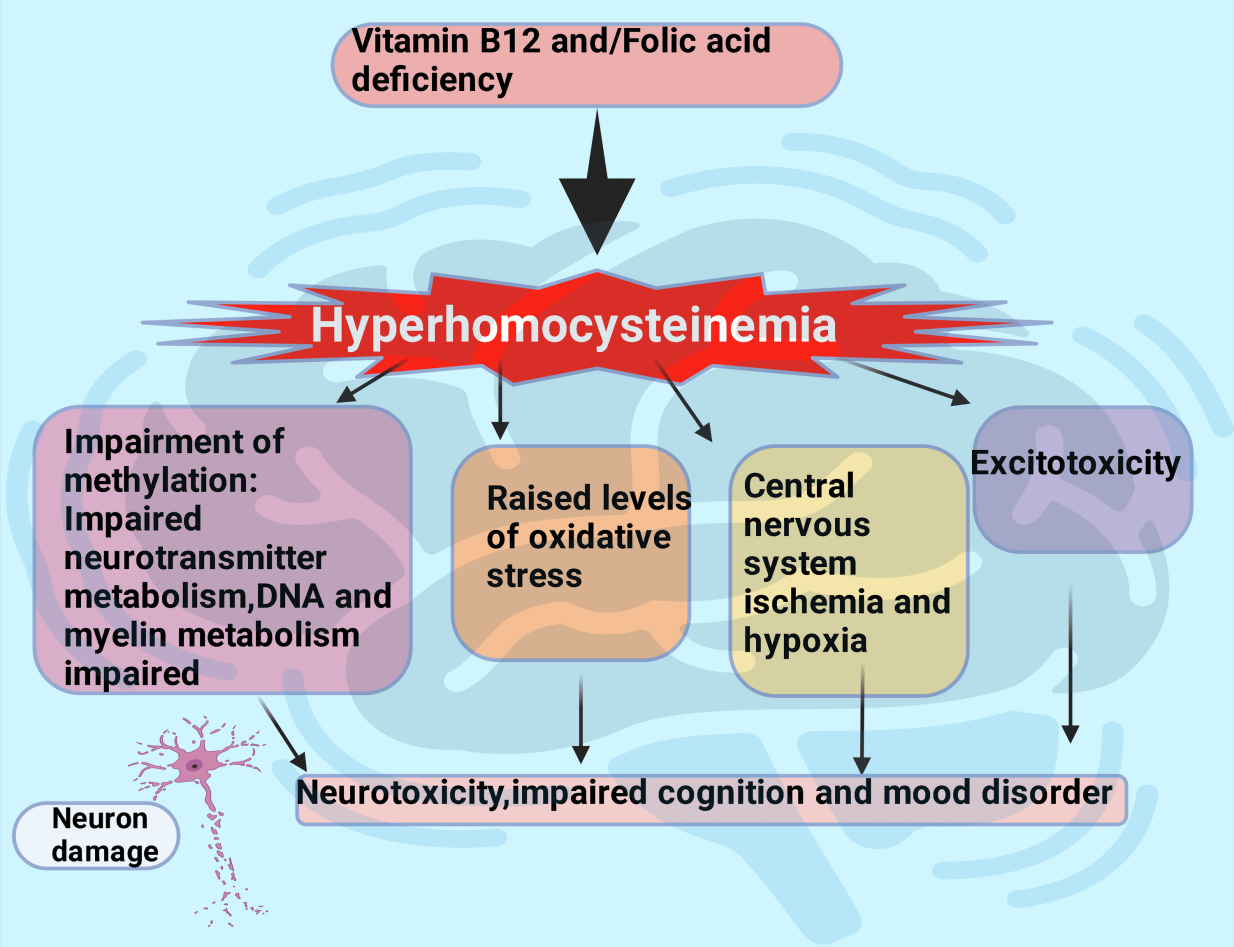
Effects of hyperhomocysteinemia on the CNS The premium version of BioRender [23] (biorender.com) was used to create this figure, accessed on March 18, 2025, under license agreement number LG281GKWV5. Illustration credit: Rahnuma Ahmad

In comparison to Group B, these results show that Group A benefited more from nutrient supplementation, with substantial increases in vitamin B₁₂ (p = 0.004) and folic acid levels (p < 0.001) and decreases in HCY (p = 0.027). The brain metabolizes HCY differently than the rest of the body, and it mostly depends on adequate levels of vitamin B₁₂ and folic acid [32]. Given that vitamin deficits have been connected to mood problems and neuropsychiatric symptoms, this improvement in vitamin status is significant [32,33]. Since excessive HCY has been linked to mental disorders like OCD and cognitive impairment (Figure 4) [14,32,34], the observed decrease in HCY levels is also noteworthy. The biochemical improvements suggest a potential metabolic shift that could contribute to alleviating OC symptoms; more investigation is required to validate these results and examine their clinical significance.

**Figure 5:**
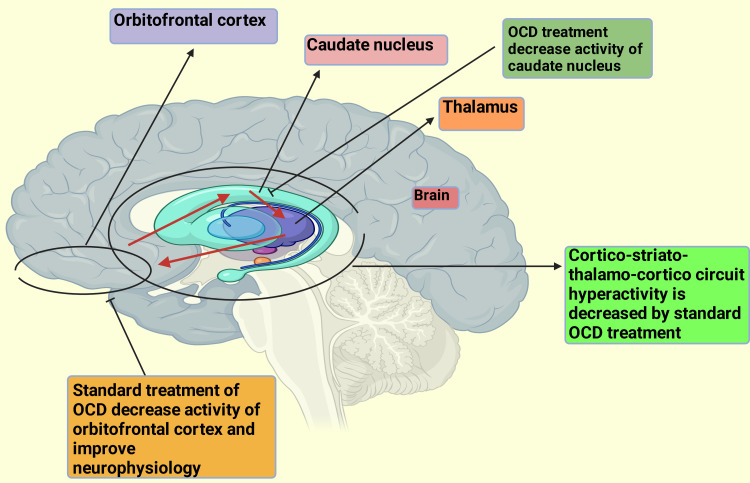
Effects of standard treatment of OCD on involved brain areas OCD, obsessive-compulsive disorder The premium version of BioRender [23] (biorender.com) was used to create this figure, accessed on March 18, 2025, under license agreement number FJ281K4EWM. Illustration credit: Rahnuma Ahmad

Changes in HCY levels are associated with improved treatment efficacy when folic acid is taken before or when paired with fluoxetine. Other antidepressants, such as imipramine, nortriptyline, and sertraline, are likewise affected by folate. S-adenosylmethionine (SAM) and folic acid may increase the efficacy of antidepressants by enhancing neurotransmitter availability and assisting with nervous system methylation processes. SAM must be produced in the brain to synthesize essential neurotransmitters like dopamine, serotonin, and noradrenaline; folate and vitamin B₁₂ are necessary for this process [32]. The two groups had no apparent differences regarding insight levels and CGI scores. On the other hand, conventional treatments for OCD partially reversed structural abnormalities and changes in functional connectivity in brain regions linked to the disorder. This suggests that conventional OCD treatment alone not only relieves symptoms but also influences the neurophysiology of the involved brain areas (Figure 5) [35].

At follow-up visits, both groups’ Y-BOCS scores decreased, indicating a decline in severe OCD symptoms. Still, there was a statistically significant difference between the groups (p = 0.046). The reduction of OC symptoms may be more pronounced when a nutrient supplement is combined with conventional treatment. Available research shows an inverse relationship between the severity of OCD and vitamin B₁₂ deficiency [36]. As previously observed, higher levels of folic acid were linked to lower Y-BOCS scores, which were inversely correlated with HCY levels. In contrast, increased HCY levels were linked with further elevated Y-BOCS scores [7,10,13]. Because low serotonin and dopamine contribute to OC symptoms and high HCY depletes folic acid, which affects neurotransmitter function, low HCY and high folic acid may help manage OCD by boosting serotonin and dopamine metabolism [6,32].

Principal findings

OCD is one of the top 10 mental disorders in which patients experience substantial distress when facing circumstances that cause difficulty. It is characterized by dysfunction in the front striatal circuit and abnormal activity in specific brain areas, which can affect the metabolism of neurotransmitters such as serotonin, dopamine, and glutamate. These dysfunctions exacerbate the emotional instability that causes individuals to engage in OC thinking. According to recent research, OCD may be treated with nutritional supplements, especially those containing folic acid and vitamin B₁₂. The purpose of this study was to see how adding these supplements affected OCD patients who had deficiencies. The group who took the supplements showed an improvement in their symptoms of OC. It is believed that increasing levels of vitamin B₁₂ and folic acid may lower HCY levels, which, in turn, may help correct the serotonin and dopamine depletion that contributes to emotional instability and OC thinking.

Also, it has been demonstrated that treating OCD improves the neurophysiology of the impacted brain regions, partially reversing anatomical abnormalities and improving functional connections within those regions. There are several obstacles to treatment, though, which result in nonadherence and a loss of follow-up. These barriers include low levels of education, being a woman, living in a rural area, poor communication systems, long follow-up periods, and the duration of therapy, especially in Bangladesh. A larger, longer-term study is needed to better examine the benefits of vitamin B₁₂ and folic acid supplementation in OCD patients. To fully understand the underlying mechanisms and genetic relationship between these nutrients and OCD and its neurophysiology, further research is also required.

Future research recommendations

More long-term studies involving a more substantial number of patients with randomized double-blinded studies may assist in synthesizing more generalizable data. This technique may offer a new perspective on additional OCD treatment, particularly for those with resistant OCD. This suggests that future research should use state-of-the-art genetic methods to investigate how changes in specific genes linked to folic acid and vitamin B₁₂ metabolism interact with environmental factors, potentially influencing OC symptoms. Such research could lead to more focused therapies and a better comprehension of the fundamental causes of OCD. Combining structural and functional imaging methods can help researchers better understand how micronutrients affect specific circuits and neurotransmitter systems in OCD patients’ brains.

Limitations

There are various restrictions on this study. Crediting the treatment for the observed improvements was challenging because a control group was absent. The small sample size and the fact that most of the samples were male may mean the results do not represent the broader OCD population. Decreased its generalizability. In addition, the brief follow-up period prompts inquiries about the long-term effects. This study’s results cannot be extended as widely because the sample was primarily male, single, and from an urban region. This study did not assess functional outcomes, such as quality of life or the impact on daily activities. Finally, other confounding variables were not considered, including genetics and other medical issues. In this study, some patients received both vitamin B₁₂ and folic acid as supplements, so whether the cumulative effect of either vitamin B₁₂ or folic acid decreased the severity of OCD also could not be determined. The study was not randomized; neither the patients nor the investigators were blinded to the intervention. This lack of blinding created a risk of reporting and measurement biases, which could potentially influence the outcomes of the intervention. It is uncertain if the nutrients’ therapeutic effects are long-lasting because OCD is a chronic neuropsychiatric disorder that can last for many years. The long-term safety of supplementation requires more investigation.

## Conclusions

The current study provides early evidence of the positive effects of nutrient supplementation (vitamin B₁₂ and folic acid) on biochemical markers and the severity of OC symptoms. This strategy may help manage OCD because it has resulted in significant increases in folic acid and vitamin B₁₂, a decrease in HCY levels, and an improvement in Y-BOCS severity scores. Future studies must confirm these findings and investigate the underlying mechanisms driving the observed improvements using larger sample sizes, control groups, and long-term follow-up. Subsequent studies with minimized limitations will yield more accurate data.
